# Stopped-Flow Spectrophotometric Study of the Kinetics and Mechanism of CO_2_ Uptake by *cis-*[Cr(C_2_O_4_)(BaraNH_2_)(OH_2_)_2_]^+^ Cation and the Acid-Catalyzed Decomposition of *cis-*[Cr(C_2_O_4_)(BaraNH_2_)OCO_2_]^−^ Anion in Aqueous Solution

**DOI:** 10.3390/molecules16097746

**Published:** 2011-09-09

**Authors:** Dagmara Jacewicz, Aleksandra Dąbrowska, Lech Chmurzyński

**Affiliations:** Department of General and Inorganic Chemistry, University of Gdańsk, Sobieskiego 18/19, 80-952 Gdańsk, Poland; Email: alex@chem.univ.gda.pl (A.D.); lech@chem.univ.gda.pl (L.C.)

**Keywords:** chromium(III) complexes, 3-aminodeoxysugar ligand, kinetics and mechanism, sodium pyruvate

## Abstract

The kinetics of CO_2_ uptake by the *cis*-[Cr(C_2_O_4_)(BaraNH_2_)(OH_2_)_2_]^+^ complex cation and the acid hydrolysis of the *cis-*[Cr(C_2_O_4_)(BaraNH_2_)OCO_2_]^−^ complex anion (where BaraNH_2_ denotes methyl 3-amino-2,3-dideoxy-β-D-*arabino*-hexopyranoside) were studied using the stopped-flow technique. The reactions under study were investigated in aqueous solution in the 288–308 K temperature range. In the case of the reaction between CO_2_ and *cis*-[Cr(C_2_O_4_)(BaraNH_2_)(OH_2_)_2_]^+^ cation variable pH values (6.82–8.91) and the constant ionic strength of solution (H^+^, Na^+^, ClO_4_^−^ = 1.0) were used. Carbon dioxide was generated by the reaction between sodium pyruvate and hydrogen peroxide. The acid hydrolysis of *cis*-[Cr(C_2_O_4_)(BaraNH_2_)OCO_2_]^−^ was investigated for varying concentrations of H^+^ ions (0.01–2.7 M). The obtained results enabled the determination of the number of steps of the studied reactions. Based on the kinetic equations, rate constants were determined for each step. Finally, mechanisms for both reactions were proposed and discussed. Based on the obtained results it was concluded that the carboxylation (CO_2_ uptake) reactions of *cis*-[Cr(C_2_O_4_)(BaraNH_2_)(OH_2_)_2_]^+^ and the decarboxylation (acid hydrolysis) of the *cis-*[Cr(C_2_O_4_)(BaraNH_2_)OCO_2_]^−^are the opposite of each other.

## 1. Introduction

The general chemistry of carbonato complexes of transition metal ions has been described by Maccoll [1] as well as Harris and co-workers [2]. The interactions of carbon dioxide with transition metal ions like CO_2_ reduction, insertion and activation have been actively pursued and the review literature on this subject is quite large [3,4,5,6,7,8]. A system consisting of transition metal ions and a bioactive organic ligand can represent a model of an enzyme and may be useful for elucidation of enzymatic reaction mechanisms. One of these classes of bioactive compounds are the aminosugars [9], which in their reactions with metal ions usually behave as monodeprotonated, bidentate ligands. Different structural factors, such as interatomic distances, bonding angles, *etc.*, lead to differences in the steric interactions of the diastereoisomers as a result of bigger or smaller distances of the carbohydrate to the different molecules, which are sometimes located in the coordination sphere of the metal ions. The amino nitrogen is the anchoring site. Subsequently, a suitable hydroxyl group deprotonates and coordinates to form a strong chelate [10]. The stabilities of complexes of various derivatives with a particular binding mode (e.g., NH_2_, O^−^) may vary by up to three orders of magnitude, depending on the relative positions of the coordinating atoms. Critical factors influencing the coordination equilibria, *i.e*., both stability and the structures of complexes, are as follows: (a) the number of the amino groups in the ligand; (b) the number of available hydroxyl functions; (c) the overall structure of the carbon chain, *i.e.*, linear or cyclic; and (d) in the case of cyclic aminosugars, the number of dioxolane rings (e.g., 1,6-anhydro-derivatives) [11]. Cyclic amino sugars, like D-glucosamine or D-mannosamine [10], form efficient but simple monomeric (NH_2_, O^−^) chelates that may differ considerably in complex stabilities from one aminosugar to another. 1,6-Anhydro derivatives using the same donor system as the parent sugars form a completely different set of species, including very unusual dimeric complexes [12]. Linear amino- alcohols also form very effective dimeric (only) species involving alkoxy-bridges, while linear diaminoalcohols also form dimeric complexes, but their binding mode is completely different from that of monoamino-derivatives. In the case of diaminoalcohols both amino groups act as anchoring sites for two metal ions. Thus, two independent {NH_2_,O−} chelates are formed, leading to dimeric complexes in which two metal ions are bound to two N-terminals of the 1,5- or 1,6-diaminoalcohol. In all cases studied both ligand conformation and absolute configuration have a distinct impact on the stabilities of the complexes formed. Studies performed for four families of aminoalcohols have shown that they are very specific chelating agents for metal ions, able to also efficiently bind metal ions in a natural environment.

In our earlier investigations two anomers of methyl 3-amino-2,3-dideoxy-D-*arabino*-hexo-pyranoside [13,14] were used as bidentate (L-L) ligands [15] to obtain two coordination compounds of general formula *cis*-[Cr(C_2_O_4_)(L-L)(OH_2_)_2_]^+^ which behave as NO_2_ biosensors. These aminodeoxysugars coordinate with chromium(III) ion through the neighboring HO-4 and 3-NH_2_ groups, which both adopt equatorial positions. Both coordinated anomers have a slightly distorted ^4^C_1_ chair conformation [13,14], compared to that of the free monosaccharide in aqueous solution. The use of these compounds in biosensors allowed us to develop a selective analytical method for the determination of the concentration of nitrogen dioxide released in biological materials [16,17].

In this paper instrumental methods of carbon dioxide (CO_2_) uptake by *cis*-[Cr(C_2_O_4_)(BaraNH_2_)(OH_2_)_2_]^+^ complex cation and acid hydrolysis of the *cis*-[Cr(C_2_O_4_)(BaraNH_2_)OCO_2_]^−^ complex anion were studied using the stopped-flow technique. Carbon dioxide was generated by the reaction between sodium pyruvate and hydrogen peroxide according to the following schematic reaction:

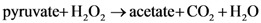
(1)


Pyruvate, by reducing the oxygen to water molecules, activates the electron transport processes in the mitochondrial respiratory chain. As a result of disturbances in this process the formation of H_2_O_2_ molecules occurs, which in a non-enzymatic reaction of pyruvate undergo an alternative conversion to CO_2_ and acetate according to reaction (1). Recent studies [18] indicate that pyruvate, under myocardial ischemia and tissue protection conditions, acts as a scavenger of free radicals resulting from oxidative stress, which in turn generates the formation of certain amounts of the hydrogen peroxide. Presumably, pyruvate by reaction with H_2_O_2_ undergoes an transformation to carbon dioxide and acetate, having under these conditions cytoprotective significance [19,20]. However, the mechanism of antioxidant action of pyruvate in this process is not completely understood, as oxygen is released in the form of free radicals.

## 2. Results and Discussion

### 2.1. Kinetics and Mechanisms of CO_2_ Uptake by *cis*-[Cr(C_2_O_4_)(BaraNH_2_)(OH_2_)_2_]^+^ Complex Cation

**Figure 1:**
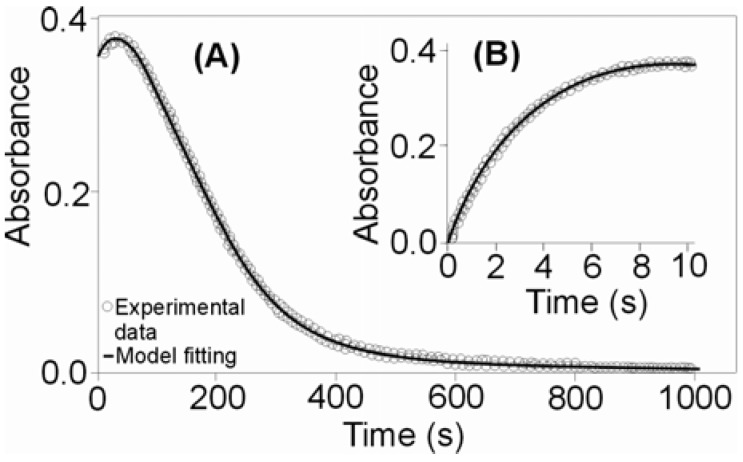
Measured time course at selected wavelength of 505 nm for the reaction of *cis*-[Cr(C_2_O_4_)(BaraNH_2_)(OH_2_)_2_]^+^ complex cation with CO_2_ ([CO_2_] = 0.01 M; pH = 6.82, T = 283K). The lines correspond to the best fit for bi-exponential (line A) and mono-exponential (line B) increases and decays.

When carrying out the kinetic measurements, it was observed that the investigated carbon dioxide uptake reaction proceeded in two steps. At the beginning of the reaction a very sharp increase in the absorbance value occurred and then, after reaching the maximum, the absorbance decreased as the reaction progressed. In the first step an intermediate product was formed, which was then transformed into the characteristic final product of the second step. The data fitting and the global value analysis of the observable rate constants for both steps were based on the consecutive reactions model. Figure 1 shows the results of fitting of the rate data to the pseudo first-order kinetic equation for the assumed consecutive reaction model (A→B→C).

**Figure 2:**
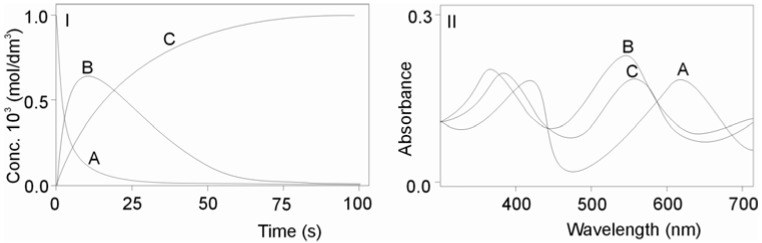
Results of the kinetic (**I**) and spectral (**II**) analysis for reactants: (**I**) Concentration curves of the decomposition of substrate A: *cis*-[Cr(C_2_O_4_)(BaraNH_2_)(OH_2_)_2_]^+^ cation and formation of product C: *cis*-[Cr(C_2_O_4_)(BaraNH_2_)(CO_3_)]^−^ anion and intermediate product B; (**II**) Absorption spectra of the reactants A, B and C.

Figure 2 presents the results of the global analysis for the reaction between carbon dioxide and the *cis*-[Cr(C_2_O_4_)(BaraNH_2_)(OH_2_)_2_]^+^ cation. The observable rate constants, for first (k_1obs_) and second steps (k_2obs_), were obtained by fitting the rate data at different temperatures and different pH values studied to the same consecutive reaction model.

#### 2.1.1. First Step for the Reaction of CO_2_ Uptake by *cis*-[Cr(C_2_O_4_)(BaraNH_2_)(OH_2_)_2_]^+^ Complex Cation

The calculations have shown that at a fixed concentration of carbon dioxide and increasing pH value the observable rate constant for CO_2_ uptake (k_1obs_) increased (Table 1) for all temperatures studied. Based on the determined acidity constants (K_1_, K_2_) (Table 2) and the observable rate constants (k_1obs_) a mathematical model for CO_2_ uptake reaction have been proposed. Consequently, after some mathematical transformation the observed pseudo first-order rate constant (k_1obs_) was obtained:

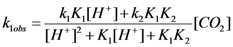
(2)


After transformation of expression (2) the following equation can be obtained:

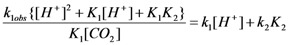
(3)


**Table 1:** Observable rate constants (k_1obs_) for the reaction of the carbon dioxide uptake by *cis*-[Cr(C_2_O_4_)(BaraNH_2_)(OH_2_)_2_]^+^ complex cation (first step) {[CO_2_] = 0.01 M, I = 1 M, NaClO_4_}.

pH	k_1obs_ [s^−1^] T = 278 K	k_1obs_ [s^−1^] T = 283 K	k_1obs_ [s^−1^] T = 288 K	k_1obs_ [s^−1^] T = 293 K	k_1obs_ [s^−1^] T = 298 K
6.82	0.12 ± 5.4E-3	0.14 ± 2.7E-3	0.15 ± 8.4E-3	0.17 ± 5.4E-3	0.19 ± 4.7E-3
7.12	0.12 ± 3.7E-3	0.15 ± 6.4E-3	0.16 ± 9.4E-3	0.18 ± 2.6E-3	0.20 ± 1.8E-3
7.45	0.13 ± 6.4E-3	0.15 ± 3.7E-3	0.16 ± 3.7E-3	0.18 ± 4.7E-3	0.21 ± 9.5E-3
7.89	0.13 ± 2.7E-3	0.16 ± 8.4E-3	0.17 ± 9.4E-3	0.19 ± 8.4E-3	0.22 ± 6.8E-3
8.22	0.15 ± 8.4E-3	0.17 ± 9.3E-3	0.18 ± 3.7E-3	0.20 ± 4.7E-3	0.24 ± 8.4E-3
8.54	0.16 ± 3.8E-3	0.18 ± 2.7E-3	0.18 ± 3.7E-3	0.21 ± 2.8E-3	0.24 ± 9.3E-3
8.92	0.17 ± 9.4E-3	0.18 ± 7.4E-3	0.19 ± 8.4E-3	0.22 ± 8.4E-3	0.25 ± 6.9E-3

**Table 2:** Rate constants (k_1_, k_2_) and acidity constants (K_1_ and K_2_) for the reaction of the carbon dioxide uptake by *cis*-[Cr(C_2_O_4_)(BaraNH_2_)(OH_2_)_2_]^+^ cation (first step) {[CO_2_] = 0.01 M, I = 1 M, NaClO_4_}.

T[K]	k_1_[s^−1^M^−1^] (pK_1_)	k_2_[s^−1^M^−1^] (pK_2_)
278	24.73 ± 0.8 (6.82 ± 0.01)	16.28 ± 0.4 (8.91 ± 0.02)
283	32.40 ± 0.7 (6.81 ± 0.02)	25.45 ± 0.9 (8.91 ± 0.03)
288	40.29 ± 1.1 (6.80 ± 0.01)	34.34 ± 0.8 (8.90 ± 0.02)
293	48.03 ± 1.3 (6.80 ± 0.01)	42.29 ± 1.2 (8.91 ± 0.03)
298	56.20 ± 1.0 (6.80 ± 0.01)	49.07 ± 1.1 (8.90 ± 0.03)
ΔH^#^	23.78 ± 0.87	16.19 ± 0.45

**Figure 3:**
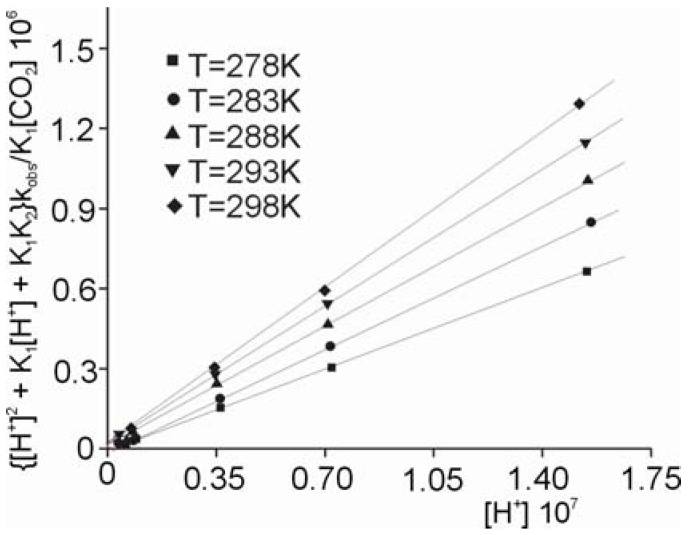
Dependence of 
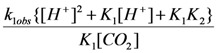
 *versus* [H^+^] for the reaction of carbon dioxide uptake by *cis*-[Cr(C_2_O_4_)(BaraNH_2_)(OH_2_)_2_]^+^ complex cation.

It turned out that the relationship between the first term (right hand) of Equation (3) and the concentration of hydrogen cation is linear for all temperatures studied, as shown in Figure 3.

Based on the relationships shown in Figure 3, the rate constants k_1_ [s^−1^ M^−1^] and k_2_ [s^−1^ M^−1^] for each temperature in the whole pH range between the measured and calculated pK_1_ and pK_2_ values were calculated. Activation enthalpies which were determined using Arrhenius’ [21] equation are collected in Table 2. The results compiled in the Table show that rate constant k_1_ [s^−1^ M^−1^] (involving the reaction of CO_2_ with the monohydroxo complex) is larger than k_2_ [s^−1^ M^−1^] (involving the reaction of CO_2_ with the dihydroxo species) at all temperatures studied.

#### 2.1.2. Second Step—The Carbonate Ring Closure

In the second step of the reaction, the final product *cis-*[Cr(C_2_O_4_)(BaraNH_2_)CO_3_]^−^ was formed from the intermediate compound. For this step only the observable rate constant k_2obs_ was determined and results are listed in Table 3. On the basis of the obtained data it can be found that at a constant temperature and increasing pH value the rate constant for the second step (k_2obs_) decreases. It can be therefore concluded that despite the existence in solution of three protolytic forms of the intermediate complex: (*cis*-[Cr(C_2_O_4_)(BaraNH_2_)(OH_2_)(OCO_2_H)]; *cis*-[Cr(C_2_O_4_)(BaraNH_2_)(OH)(OCO_2_H)]^−^ and *cis*-[Cr(C_2_O_4_)(BaraNH_2_)(OH)(OCO_2_)]^2−^), the ring closure occurs more readily for only one of them, namely *cis*-[Cr(C_2_O_4_)(BaraNH_2_)(OH_2_)(OCO_2_H)]. Presumably, the substitution of water molecules in the neutral complex *cis-*[Cr(C_2_O_4_)(BaraNH_2_)(OH_2_)(OCO_2_H)]^0^ occurs more easily than in the case of the monoanion complex ion, in which the central atom is linked to a carbonate ligand with two oxygen atoms. The ring closure step is much slower than the first step of CO_2_ uptake and therefore it determines the rate of the process.

**Table 3:** The observable rate constants k_2obs_ [s^−1^] for the ring closure of *cis*-[Cr(C_2_O_4_)(BaraNH_2_)(OH_2_)_2_]^+^ complex cation.

pH	k_2obs_ [s^−1^] T = 278 K	k_2obs_ [s^−1^] T = 283 K	k_2obs_ [s^−1^] T = 288 K	k_2obs_ [s^−1^] T = 293 K	k_2obs_ [s^−1^] T = 298 K
6.82	1.61E-3 ± 6.4E-5	1.86E-3 ± 7.5E-5	2.35E-3 ± 4.3E-5	3.11E-3 ± 8.5E-5	4.78E-3 ± 8.4E-5
7.12	1.36E-3 ± 6.4E-5	1.58E-3 ± 8.4E-5	1.93E-3 ± 2.5E-5	2.39E-3 ± 3.5E-5	3.32E-3 ± 3.6E-5
7.45	1.19E-3 ± 7.3E-5	1.35E-3 ± 4.6E-5	1.59E-3 ± 7.4E-5	1.93E-3 ± 6.4E-5	2.46E-3 ± 2.6E-5
7.89	1.06E-3 ± 8.4E-5	1.24E-3 ± 2.5E-5	1.39E-3 ± 8.4E-5	1.64E-3 ± 2.5E-5	2.01E-3 ± 1.8E-5
8.22	9.81E-4 ± 3.6E-6	1.08E-3 ± 5.3E-5	1.22E-3 ± 4.3E-5	1.41E-3 ± 7.5E-5	1.67E-3 ± 3.4E-5
8.54	1.04E-4 ± 2.8E-6	1.19E-3 ± 8.4E-5	1.37E-3 ± 2.6E-5	1.61E-3 ± 3.5E-5	1.98E-3 ± 2.7E-5
8.92	8.06E-4 ± 7.4E-6	8.83E-4 ± 1.7E-6	9.68E-4 ± 8.4E-6	1.08E-3 ± 5.6E-5	1.24E-3 ± 8.4E-5

#### 2.1.3. Proposed Mechanism of Carbon Dioxide Uptake by *cis*-[Cr(C_2_O_4_)(BaraNH_2_)(OH_2_)_2_]^+^ Complex Cation

**Scheme 1:**
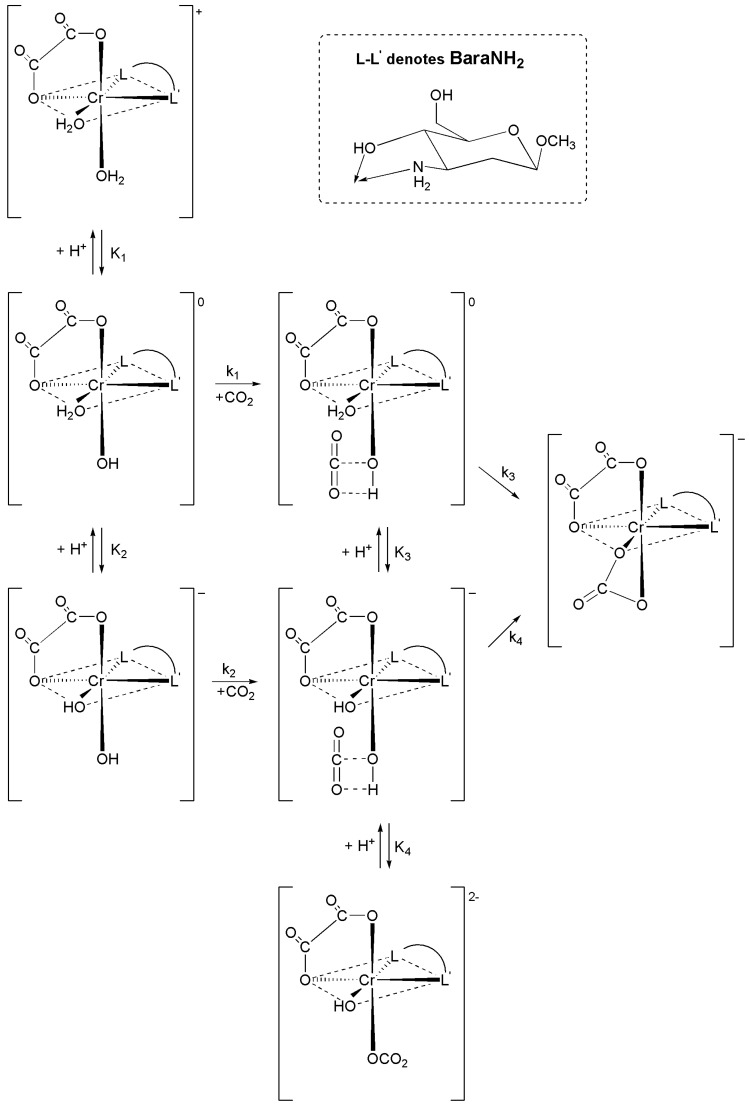
Proposed mechanism of the two step reaction between CO_2_ and *cis*-[Cr(C_2_O_4_)(BaraNH_2_)(OH_2_)_2_]^+^ complex cation.

On the basis of the results of kinetic measurements of the CO_2_ uptake by the *cis*-[Cr(C_2_O_4_)(BaraNH_2_)(OH_2_)_2_]^+^ cation a mechanism of this reaction has been proposed and presented in Scheme 1. The coordination cation (*cis*-[Cr(C_2_O_4_)(BaraNH_2_)(OH_2_)_2_]^+^) exists in solution in three protolytic forms: (*cis*-[Cr(C_2_O_4_)(BaraNH_2_)(OH_2_)_2_]^+^; *cis*-[Cr(C_2_O_4_)(BaraNH_2_)(OH)(OH_2_)] and *cis*-[Cr(C_2_O_4_)(BaraNH_2_)(OH)_2_]^−^ whose concentrations are determined by the values of the acidity constants, K_1_ and K_2_. As the pH increases the concentration of the protolytic form containing two hydroxyl groups increases. In the first (fast) step the carbon dioxide uptake reaction occurs and intermediate species (*cis-*[Cr(C_2_O_4_)(BaraNH_2_)(OH_2_)(OCO_2_H)]^0^; *cis-*[Cr(C_2_O_4_)(BaraNH_2_)(OH)(OCO_2_H)]^−^; *cis-*[Cr(C_2_O_4_)(BaraNH_2_)(OH)(OCO_2_)]^2−^) are formed. In this step the carbonate (or bicarbonate) anion is linked to the chromium(III) cation by one oxygen atom. The carbon dioxide uptake reaction occurs very fast since during this process no breakage of a metal–oxygen (from the hydroxyl group) bond occurs. A new bond between the carbon atoms of carbon dioxide and the oxygen atoms of the hydroxyl group of the complex ion was created [22,23,24,25,26], so it is seen that hydrogen bonding plays an important role. In this step three intermediate species exist in solution, whose concentrations are determined by the values of the acidity constants K_3_ and K_4_. In the second step the final product, *cis*-[Cr(C_2_O_4_)(BaraNH_2_)CO_3_]^−^ anion, is formed. This step is disturbed by the hydrolysis reaction of the anionic product [27,28]. Due to this fact the acidity constants K_3_, K_4_ and the rate constants k_3_ and k_4_ cannot be determined.

### 2.2. Acid Hydrolysis of the *cis*-[Cr(C_2_O_4_)(BaraNH_2_)OCO_2_]^−^ Complex Anion

The decarboxylation reaction (the release of CO_2_ from the *cis-*[Cr(C_2_O_4_)(BaraNH_2_)OCO_2_]^−^ anion) as a reaction opposite to the reaction of the carbon dioxide uptake by the *cis*-[Cr(C_2_O_4_)(BaraNH_2_)(OH_2_)_2_]^+^ ion also occurs in two steps. It has been confirmed by the shape of approximated curve of the absorption *versus* time dependence, which rises and falls biexponentially as in the case of the uptake reaction described in Section 2.1. In the first step (the opening of the carbonate ring preceded by the hydration process the intermediate product *cis-*[Cr(C_2_O_4_)(BaraNH_2_)(OH_2_)(OCO_2_H)]^0^ is formed, and then, in the second (fast) step is transformed to the final product, *cis-*[Cr(C_2_O_4_)(BaraNH_2_)(OH_2_)]^+^ cation. The observable rate constants for the first (k_1obs_) and second step (k_2obs_) of the acid-hydrolysis of the *cis-*[Cr(C_2_O_4_)(BaraNH_2_)(OCO_2_)]^−^ ion were determined for the pseudo-first order reaction in the consecutive (A→B→C) reaction model and are listed in Table 4 and Table 5. It can be found that at a constant temperature and increasing [H^+^] the rate constant k_1obs_ for the first step increases. On the other hand, the observable rate constants k_2obs_ in this same conditions does not change.

**Table 4:** Relationship between the pseudo-first-order rate constants (k_1obs_) and the H^+^ cation concentration for the acid-catalyzed hydrolysis of the *cis*-[Cr(C_2_O_4_)(BaraNH_2_)OCO_2_]^−^ complex anion.

	k_1obs_ [s^−1^]				
[c_*HCLO*_4__]	T = 278 K	T = 283 K	T = 288 K	T = 293 K	T = 298 K
0.01	2.08 ± 0.02	2.53 ± 0.08	2.99 ± 0.02	3.53 ± 0.05	4.05 ± 0.04
0.05	4.00 ± 0.03	4.54 ± 0.05	5.29 ± 0.05	6.33 ± 0.04	6.45 ± 0.06
0.1	4.86 ± 0.06	5.05 ± 0.09	5.62 ± 0.04	6.71 ± 0.09	6.92 ± 0.02
0.3	4.95 ± 0.08	5.51 ± 0.08	5.82 ± 0.08	6.77 ± 0.02	7.27 ± 0.05
0.5	5.00 ± 0.02	5.52 ± 0.09	6.34 ± 0.07	6.83 ± 0.05	7.32 ± 0.03
0.8	5.02 ± 0.06	5.53 ± 0.04	6.35 ± 0.03	6.84 ± 0.06	7.35 ± 0.05
1.1	5.03 ± 0.04	5.54 ± 0.06	6.36 ± 0.06	6.85 ± 0.02	7.36 ± 0.02
1.4	5.03 ± 0.09	5.54 ± 0.03	6.37 ± 0.02	6.86 ± 0.08	7.37 ± 0.03
1.7	5.04 ± 0.01	5.55 ± 0.02	6.38 ± 0.08	6.87 ± 0.04	7.38 ± 0.04
2.0	5.05 ± 0.03	5.56 ± 0.05	6.39 ± 0.03	6.88 ± 0.02	7.39 ± 0.07
2.3	5.05 ± 0.02	5.57 ± 0.07	6.40 ± 0.08	6.89 ± 0.02	7.39 ± 0.05
2.7	5.06 ± 0.05	5.58 ± 0.01	6.42 ± 0.02	6.89 ± 0.06	7.39 ± 0.06

**Table 5:** Calculated rate constants (k_1_, k_2_) and protonation constant (K) of the *cis*-[Cr(C_2_O_4_)(BaraNH_2_)OCO_2_]^−^ complex anion.

T[K]	k_1_ [s^−1^]	k_2_ [s^−1^] = k_2obs_	K [M^−1^]
278	5.10 ± 0.09	5.42 ± 0.09	1.14 ± 0.02
283	5.62 ± 0.08	6.47 ± 0.08	1.15 ± 0.01
288	6.29 ± 0.09	7.52 ± 0.08	1.15 ± 0.02
293	6.92 ± 0.08	8.47 ± 0.07	1.14 ± 0.02
298	7.43 ± 0.09	9.53 ± 0.09	1.14 ± 0.03

Major features resulting this analysis were (Equation 5) the independence of k_2obs_ on [H^+^] and (Equation 8 in Experimental) the substantial acid dependence of k_1obs_, which fits the rate expressions:

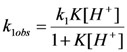
(4)

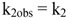
(5)


**Figure 4:**
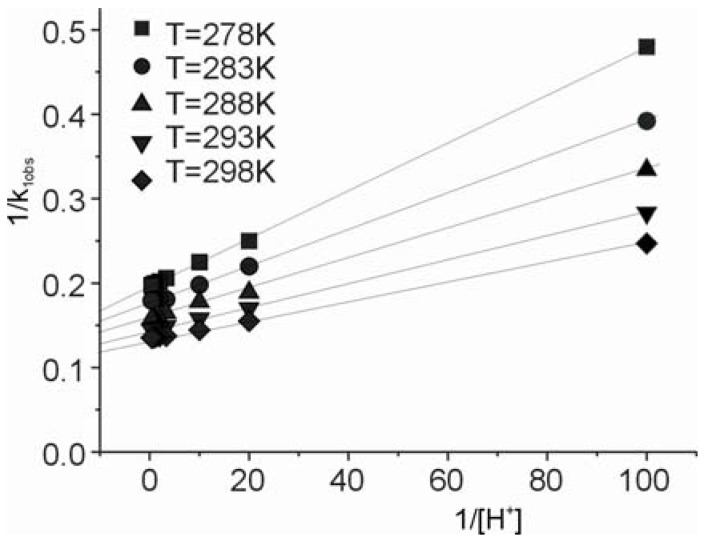
Dependence of 1/k_1obs_* versus* 1/[H^+^] for the hydrolysis of the *cis-*[Cr(C_2_O_4_)(BaraNH_2_)OCO_2_]^−^ ion in HClO_4_ solution for all temperatures studied.

The relationship between the 1/k_1obs_ and 1/[H^+^] values is linear for all temperatures studied, as shown in Figure 4.

Using the linear dependence shown in Figure 4 and Equation (5), the rate constants (k_1_ [s^−1^] and k_2_ [s^−1^]), whose values are summarized in Table 5, have been determined. As shown in Table 5, both rate constants increase with the increasing temperature. On the other hand, the constant K ~ 1.14 [M^−1^] (Table 5) describing the protonation equilibrium between the *cis*-[Cr(C_2_O_4_)(BaraNH_2_)(OCO_2_)]^−^ complex anion and *cis*-[Cr(C_2_O_4_)(BaraNH_2_)(OCO_2_H)]^0^ neutral complex has the same value at each temperature studied. This value suggests that some 50% of the chelate should exist in the protonated form in acid solution.

#### 2.2.1. Proposed Mechanism for the Acid-Catalyzed Decomposition of *cis*-[Cr(C_2_O_4_)(BaraNH_2_)OCO_2_]^−^ Complex Anion in Aqueous Solution

**Scheme 2:**
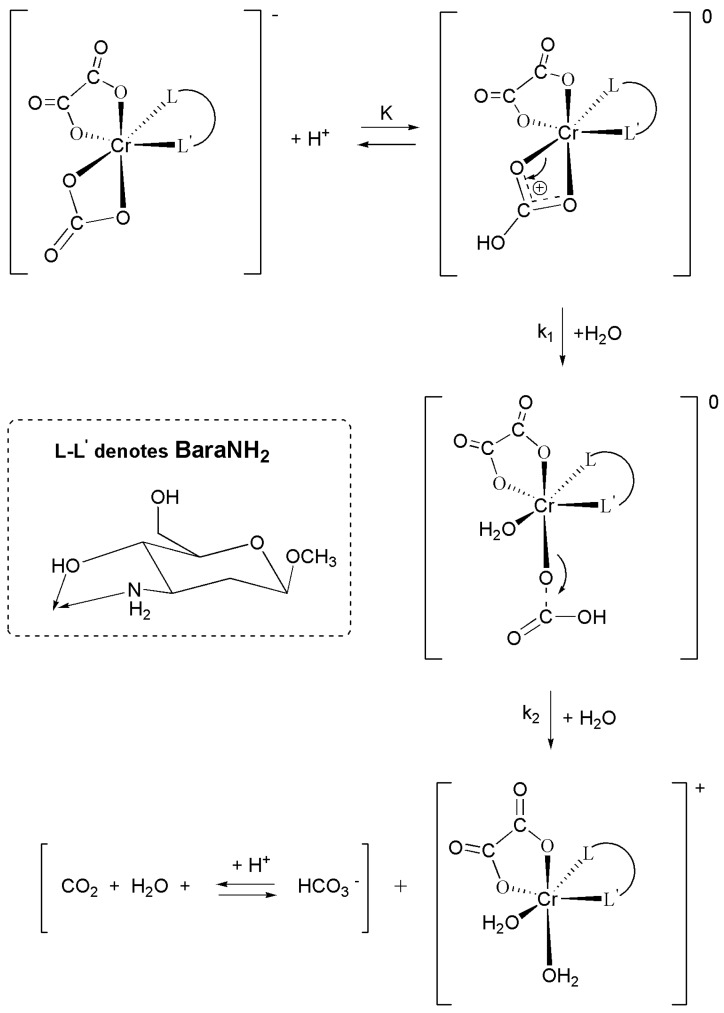
Proposed mechanism of the hydrolysis of the *cis*-[Cr(C_2_O_4_)(BaraNH_2_)OCO_2_]^−^ complex anion.

On the basis of the kinetic measurements performed, a mechanism of the hydrolysis reactions catalyzed by H^+^ ions for the complex of chromium(III) (with the oxalate and the carbonate anions as well as the aminosugar as ligands) can be proposed (Scheme 2). The acid-catalyzed decomposition of *cis*-[Cr(C_2_O_4_)(BaraNH_2_)OCO_2_]^−^ ion in aqueous solution can be summarized by the following equation:

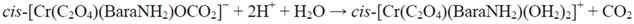
(6)


As shown in Scheme 2 the reaction studied proceeds in two steps. The coordination ion (*cis-*[Cr(C_2_O_4_)(BaraNH_2_)OCO_2_]^−^) exists in solution in equilibrium with its neutral form *cis-*[Cr(C_2_O_4_)(BaraNH_2_)(OCO_2_H)]^0^ described by acidity constant K_a_ = 1/K. The first step of the reaction, in which the carbonate ring is opened, is slow. The constant K describes the protonation equilibrium of the [Cr(C_2_O_4_)(BaraNH_2_)OCO_2_]^−^ anion which is established prior to the two-step hydrolysis. The first step of the reaction, in which the carbonate ring is opened, is slow. This step is dependent on the concentration of hydrogen cations. With the increased temperature the rate constant k_1_[s^−1^] is increasing, consequently, the reaction rate increases. A significant increase in the value of the rate constant of the chelate ring opening with increasing concentration of hydrogen ions is related to the protonation of CO_3_^2−^ group and the subsequent breaking of M–OCO_2_H bond and the following substitution in one coordination site of the water molecule, which is present in an aqueous environment of this reaction. The second step is much faster than the first one—This is the hydration reaction. The rate constant of this step is not dependent on the concentration of hydrogen cations in the whole temperature range, however, depends on the temperature. The independence of the rate of the second step (hydration reaction) on the concentration of the hydrogen ion is due to the fact that it is a reaction in which the bicarbonate ligand is exchanged with a molecule of water. This exchange occurs much faster at higher temperatures, hence the temperature dependence for the second step can be determined.

## 3. Experimental

### 3.1. Reagents

All the reagents required for the synthesis were purchased from Sigma (Poznań, Poland).

### 3.2. Synthesis of Methyl 3-amino-2,3-dideoxy-β-D-arabinohexopyranoside (BaraNH_2_)

BaraNH_2_ was synthesized according to the procedures described in [14].

### 3.3. Synthesis of *cis*-[Cr(C_2_O_4_)(BaraNH_2_)(OH_2_)_2_]^+^

In the first step the *cis*-K[Cr(C_2_O_4_)_2_(OH_2_)_2_]·3H_2_O was synthesized according to the procedures described in [29]. Next, a solution (40 mL) of *cis*-K[Cr(C_2_O_4_)_2_(OH_2_)_2_]·3H_2_O (1.96 g) in water was heated for 15 min at a temperature of 338–343 K. The pH of the solution was adjusted to *ca*. 9; to give a dark green colour. To this mixture was then added a stoichiometric quantity of methyl 3-amino-2,3-dideoxy-β-D-*arabino*-hexopyranoside (5 mmol), dissolved in water (10 mL, pH ≈ 9). The resulting solution was stirred for *ca*. 15 min, cooled, and then acidified with 0.5 M HClO_4_ to pH ≈ 2. The anionic product (*cis*-[Cr(C_2_O_4_)_2_(BaraNH_2_)]^−^) was isolated by ion-exchange column chromatography, with a packing of strongly basic DOWEX 1 × 8 anion exchanger. Ferric nitrate (0.2 M, 25 mL) and nitric acid (2 M, 15 mL) were added to the solution (180 mL) of the *cis*-[Cr(C_2_O_4_)_2_(BaraNH_2_)]^−^ complex. This mixture was then heated for 25 min at 320 K. To separate the product—The *cis*-[Cr(C_2_O_4_)(BaraNH_2_)(OH_2_)_2_]^+^ ion—the post-reaction mixture was transferred to a chromatographic column for gradient elution. Stock solution of complex ion was obtained by freezing. The molar ratio of Cr(III):C_2_O_4_^2−^:BaraNH_2_:H_2_O was 1:1:1:2. These results are consistent with the formula *cis*-[Cr(C_2_O_4_)(BaraNH_2_)(OH_2_)_2_]^+^. The composition of this complex ion was determined by chromium, oxalate and sugar residue analyses. Chromium(III) was determined spectrophotometrically as CrO_4_^2^^-^ at 372 nm after oxidizing by H_2_O_2_ in an alkaline medium. Oxalate was determined manganometrically after separation from the BaraNH_2_ and Cr(III). The BaraNH_2_ sugar residue was determined spectrophotometrically as a free ligand after liberation from the complex ion *cis*-[Cr(C_2_O_4_)(BaraNH_2_)(OH_2_)_2_]^+^. For this purpose a solution of the complex in 0.1 M HClO_4_ with 0.01 M Cr(II) was heated for 20 min at 50 °C under argon. Then chromium(II) was oxidized and a solution was diluted with 0.1 M HClO_4_. Concentration of BaraNH_2_ was calculated from absorbances at 312 nm and 265 nm using molar absorption coefficient of 14 M^-1^ cm^-1^ and 27 M^-1^ cm^-1^, respectively.

### 3.4. Synthesis of the *cis*-[Cr(C_2_O_4_)(BaraNH_2_)OCO_2_]^−^

A solution of K_2_CO_3_ (40 mg, 10 mL) was gradually added to a solution of *cis*-[Cr(C_2_O_4_) (BaraNH_2_)(OH_2_)_2_]^+^ ion in water (1 g, 10 mL) until the pH reached 8.5. The solution obtained was stirred for 10 min, then cooled to 273 K. The final product, *cis*-K[Cr(C_2_O_4_)(BaraNH_2_)OCO_2_], precipitated in the form of a dark blue powder which was separated from the solution, washed with ethanol and recrystallized from hot water. The percentage compositions calculated for the empirical formula of C_10_H_15_CrNO_11_K: C, 28.8; H, 3.6; O, 42.3 are in agreement with the data obtained from elemental analysis: C, 28.7 H, 3.6; O, 42.4. In the solutions the respective molar ratios Cr(III):C_2_O_4_^2−^:BaraNH_2_: CO_3_^2−^ in the compound under study were: 1:1:1:1, where the chromium(III) ion and methyl 3-amino-2,3-dideoxy-β-D-*arabino*-hexopyranoside in the *cis-*[Cr(C_2_O_4_)(BaraNH_2_) OCO_2_]^−^ coordination ion were determined as described above and the content of the carbonate anion was determined quantitatively by acid-base titration using a standard solution of 0.112 M HCl in the presence of 1% aq. methyl orange.

### 3.5. Spectral Measurements

Spectral measurements were carried out in the UV-Vis region using a Perkin-Elmer Lambda 650 spectrophotometer equipped with a Peltier temperature control system. The system features high heating and cooling rates and excellent temperature accuracy, which is an essential requirement for measurements. The instrument has the scan accuracy of 1 nm and 1 nm slit width at a scanning rate of 120.00 nm min^−1^.

### 3.6. Determination of Acidity Constants of *cis*-[Cr(C_2_O_4_)(BaraNH_2_)(OH_2_)_2_]^+^ Complex Ion

Samples to spectrophotometric measurements were prepared immediately before recording the spectra. Thus, a aqueous solution of the *cis*-[Cr(C_2_O_4_)(BaraNH_2_)(OH_2_)_2_]^+^ (1.5 mL, 0.01 M) was mixed with an equal volume of appropriate buffer solution Tris [*tris-*(hydroxymethyl)-aminomethane)]. pH measurements were made with a CX 731 pH-meter (reading accuracy of 0.01 pH unit) and a combined electrode manufactured by Hanna. The pK values for the acid dissociation of *cis*-[Cr(C_2_O_4_)(BaraNH_2_)(OH_2_)_2_]^+^ were determined spectrophotometrically over 340/700 nm range. Then, the pK_1_ and pK_2_ values in the ground state were computed using Origin 8.5 program, based on absorbance variations at a selected wavelength and applying the non-linear least squares method according to the Equation (7) [30]:

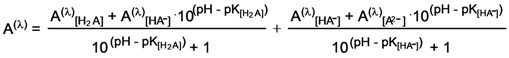
(7)
where [H_2_A], [HA^-^] and [A^2^^-^] denote *cis*-[Cr(C_2_O_4_)(BaraNH_2_)(OH_2_)_2_]^+^, *cis*-[Cr(C_2_O_4_)(BaraNH_2_) (OH_2_)(OH)] and *cis*-[Cr(C_2_O_4_)(BaraNH_2_)(OH)_2_]^-^, respectively, at a particular wavelength l. Absorption spectra in the UV-visible region were measured using a Perkin-Elmer Lambda 650 spectrophotometer.

### 3.7. Determination of Acidity Constant for the *cis*-[Cr(C_2_O_4_)(BaraNH_2_)OCO_2_]_-_ Complex Ion

The zero absorbance time (A_obs_) for the reacting solution was determined by extrapolation of A_t_ to t = 0 with a dead stirring time of 2 ms. The protonation constant K was obtained from relationship (8) described by Buckingham *et al.* [28] as a plot of (A_1_ − A_obs_) *vs*. [H^+^] with the use of the Origin 8.5. program:

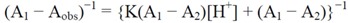
(8)
where A_1_ represents the absorbance of the complex ion *cis-*[Cr(C_2_O_4_)(BaraNH_2_)OCO_2_]^−^ in the absence of acid (ionic strength = 1.0 M obtained with NaClO_4_), and A_2_ represents the absorbance of the bicarbonate chelate *cis-*[Cr(C_2_O_4_)(BaraNH_2_)OCO_2_H]. The acidity constant was marked using an Applied Photophysics SX-17 MV stopped-flow spectrophotometer.

### 3.8. Kinetic Measurements for the Reaction of CO_2_ Uptake by *cis*-[Cr(C_2_O_4_) (BaraNH_2_)(OH_2_)_2_]_+_

The CO_2_ uptake reaction [22] by *cis*-[Cr(C_2_O_4_)(BaraNH_2_)(OH_2_)_2_]^+^ ion was investigated using an Applied Photophysics SX-17 MV stopped–flow spectrophotometer. Carbon dioxide uptake reactions were studied at a constant ionic strength of 1 M (NaClO_4_) keeping [CO_2_] >> [total Cr]) and over the pH and temperature ranges: 6.81 < pH < 8.91 and 278 K < T < 298 K, respectively. The measurements were carried out at five temperatures (278, 283, 288, 293 and 298 K) and at constant concentration of carbon dioxide (0.01 M). Carbon dioxide was generated by the reaction between sodium pyruvate and hydrogen peroxide according to the Equation (1). The solutions of the complex ion were prepared by mixing *cis*-[Cr(C_2_O_4_)(BaraNH_2_)(OH_2_)_2_]^+^ (0.5 mL, 10^−3^ M) with Tris buffer (2 mL, 0.2 M) and NaClO_4_ (2 mL, 2 M) solutions. The reactions were monitored at wavelengths, which offer the largest absorbance difference between reactant and product. The observed pseudo-first-order rate constants were calculated by using a ‘‘Glint’’ program based on global analysis [31,32] and were reported as the mean of at least four kinetic runs.

### 3.9. Kinetic Measurements for the Reaction of Acid-Catalyzed Decomposition of *cis*-[Cr(C_2_O_4_)(BaraNH_2_)OCO_2_]^−^

The decarboxylation reaction was investigated [24] using an Applied Photophysics SX-17 MV stopped-flow spectrophotometer. In order to carry out kinetic studies of the acid-catalysed hydrolysis of the *cis*-[Cr(C_2_O_4_)(BaraNH_2_)OCO_2_]^−^ coordination ion, eleven standard solutions of HClO_4_ were prepared. Their final concentrations were: 0.01, 0.05, 0.1, 0.3, 0.5, 0.8, 1.1, 1.4, 1.7, 2.0, 2.3 and 2.7 M.

## 4. Conclusions

In this paper, the kinetics and mechanisms of CO_2_ uptake by *cis-*[Cr(C_2_O_4_)(BaraNH_2_)(OH_2_)_2_]^+^ complex cation and acid-catalyzed decomposition of *cis-*[Cr(C_2_O_4_)(BaraNH_2_)OCO_2_]^−^ complex anion in aqueous solution have been studied. Kinetic investigation by the spectrophotometic stopped-flow technique was applied to track the progress of these processes. The uptake reaction of carbon dioxide by *cis-*[Cr(C_2_O_4_)(BaraNH_2_)(OH_2_)_2_]^+^ indicates unambiguously that it occurs in two steps: Addition of the molecule of carbon dioxide to complex cation (first quick step-CO_2_ uptake) is the first one, and the subsequent creation of the bidentate carbonate ion (second step—Ring closure) in the next step. The second one is about 10 times slower than the first step due to the fact that it consists of breaking of the chromium-oxygen bond and the subsequent creation of the new bond with the carbon dioxide.

The second part of our studies aimed at establishing the mechanism of the decarboxylation reaction (the release of carbon dioxide from the *cis*-[Cr(C_2_O_4_)(BaraNH_2_)OCO_2_]^−^ complex anion) as the “opposite” to the CO_2_ uptake reaction of *cis-*[Cr(C_2_O_4_)(BaraNH_2_)(OH_2_)_2_]^+^ complex cation. The acid-catalyzed decomposition of *cis*-[Cr(C_2_O_4_)(BaraNH_2_)OCO_2_]^−^ proved to be the biphasic process following a rapid preprotonation reaction. We have found differences in the observed rate constants for four-membered bicarbonate chelate ring opening (k_1obs_ = k_slow_) and spontaneous hydrolysis of the monodentate *cis-*[Cr(C_2_O_4_)(BaraNH_2_)(OH_2_)(OCO_2_H)] intermediate (k_2obs_ = k_fast_).

Based on the obtained data it can be concluded that the carboxylation (the CO_2_ uptake) of *cis-*[Cr(C_2_O_4_)(BaraNH_2_)(OH_2_)_2_]^+^ complex cation and the decarboxylation (the acid hydrolysis) of *cis-*[Cr(C_2_O_4_)(BaraNH_2_)OCO_2_]^−^ complex anion are reactions opposite to each other, as is illustrated by the simplified equation (9) shown below:

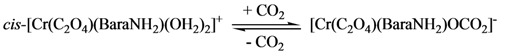
(9)


## References

[1] Maccoll P., Carlos R. (1969). Carbonato complexes of cobalt(III). Coord. Chem. Rev..

[2] Harris G.M., Krishnamurthy K.V., Sastri V.S. (1970). Chemistry of the metal carbonato complexes. Chem. Rev..

[3] Min D., Lee S.W. (2002). Terbium-oxalate-pyridinedicarboxylate coordination polymers suggesting the reductive coupling of carbon dioxide (CO_2_) to oxalate (C_2_O_4_^2−^):[Tb_2_(3,5-PDC)_2_(H_2_O)_4_(C_2_O_4_)]·2H_2_O and [Tb(2,4-PDC)(H_2_O)(C_2_O_4_)_0.5_] (PDC = pyridinedicarboxylate). Inorg. Chem. Commun..

[4] Fujita E. (1999). Photochemical carbon dioxide reduction with metal complexes. Coord. Chem. Rev..

[5] Yin X., Moss J.R. (1999). Recent developments in the activation of carbon dioxide by metal complexes.

[6] Ni J., Qiu Y., Cox T.M., Jones C.A., Berry C., Melon L., Bott S. (1996). Carbon dioxide chemistry: Characterization of the carbon dioxide reaction product of a dinuclear titanium complex. Organomet.

[7] Hanna T.A., Baranger A.M., Bergman R.G. (1995). Reaction of carbon dioxide and heterocumulenes with an unsymmetrical metal-metal bond. Direct addition of carbon dioxide across a zirconium-iridium bond and stoichiometric reduction of carbon dioxide to formate. J. Am. Chem. Soc..

[8] Antiñolo A., Fajardo M., García-Yuste S., del Hierro I., Otero A., Elkrami S., Mourad Y., Mugnier Y. (1995). Synthesis, electrochemistry and reactivity of formato- and acetate-niobocene complexes. J. Chem. Soc. Dalton Trans..

[9] Saotome C., Ono M., Akita H. (2000). Chemoenzymatic syntheses of *N*-trifluoroacetyl-L-daunosamine, *N*-trifluoroacetyl-L-acosamine, *N*-benzoyl-D-acosamine and *N*-benzoyl-D-ristosamine from an achiral precursor, methyl sorbate. Tetrahedron: Asymmetry.

[10] Kozłowski H., Decock P., Olivier I., Micera G., Pusino A., Pettit L.D. (1990). Stability and structure of copper(II) complexes with 2-amino-2-deoxy-D-mannose and some derivatives. Carbohydr. Res..

[11] Jeżowska-Bojczuk M., Kozłowski H., Decock P., Cerny M., Trnka T. (1992). Potentiometric and spectroscopic studies of the binding of copper(II) ions by aminodeoxy derivatives of 1,6-anhydro-β-D-glucopyranose. Carbohydr. Res..

[12] Jeżowska-Bojczuk M., Kozłowski H., Trnka T., Cerny M. (1994). Interaction of 1,6-anhydro derivatives of amino sugars with copper(II) ions. Carbohydr. Res..

[13] Dąbrowska A., Sikorski A., Jacewicz D., Chmurzyński L. (2004). X-ray and conformational analysis of methyl 3-amino-2,3-dideoxy-α-D-*arabino*-hexopyranoside. Carbohydr. Res..

[14] Dąbrowska A., Sikorski A., Jacewicz D., Chmurzyński L. (2005). Crystal structure of methyl 3-amino-2,3-dideoxy-β-D-*arabino*-hexopyranoside. Stabilization of the crystal lattice by a double network of N-H^…^O (O-H^…^N) and C-H^…^O interactions. Carbohydr. Res..

[15] Dąbrowska A., Jacewicz D., Makowska J., Makowski M., Chmurzyński L. (2005). Ab initio study of the energetics of protonation and deprotonation of the methyl 3-amino-2,3-dideoxyhexopyranosides isomers. J. Mol. Struct. Theochem.

[16] Dąbrowska A., Jacewicz D., Łapińska A., Banecki B., Figarski A., Szkatuła M., Lehman J., Krajewski J., Kubasik-Juraniec J., Woźniak M. (2005). Pivotal participation of nitrogen dioxide in L-arginine induced acute necrotizing pancreatitis; protective role of superoxide scavenger 4-OH TEMPO. Biochem. Biophys. Res. Commun..

[17] Jacewicz D., Dabrowska A., Wyrzykowski D., Pranczk J., Wozniak M., Kubasik-Juraniec J., Knap N., Siedlecka K., Neuwelt A.J., Chmurzynski L. (2010). A novel biosensor for evaluation of apoptotic or necrotic effects of nitrogen dioxide during acute pancreatitis in rat. Sensors.

[18] Cavallini L., Valente M., Rigobello M.P. (1990). The protective action of pyruvate on recovery of ischemic rat heart: Comparison with other oxidizable substrates. J. Mol. Cell. Cardiol..

[19] Bunger R., Swindall B., Brodie D., Zdunek D., Stiegler H., Walter G. (1986). Pyruvate attenuation of hypoxia damage in isolated working guinea-pig hart. J. Mol. Cell. Cardiol..

[20] Mentzer R.M., Van Wylen D.G.L., Shodi J. (1989). Effect of pyruvate on regional ventricular function in normal and stunned myocardium. Ann. Surg..

[21] Helferich F.G. (2001). Kinetics of Homogeneous Multistep Reactions.

[22] Chaffee E., Dasgupta T.P., Harris G.M. (1973). Kinetics and mechanism of aquation and formation reactions of carbonato complexes. V. Carbon dioxide uptake by hydroxopentaamminecobalt(III) ion to form carbonatopentaamminecobalt(III) ion. J. Am. Chem. Soc..

[23] Dasgupta T.P., Harris G.M. (1977). Kinetics and mechanism of aquation and formation reactions of carbonato complexes. 11. Carbon dioxide uptake and intramolecular carbonato ligand chelation in aqueous solution of *cis*- and *trans*-diaquo(1,4,8,11-tetraazacyclotetradecane)cobalt(III) cations. J. Am. Chem. Soc..

[24] Dasgupta T.P., Harris G.M. (1974). Kinetics and mechanism of aquation and formation reactions of carbonato complexes. VII. Acid-catalyzed aquation of carbonato(nitrilotriacetato)cobaltate(III) ion. Inorg. Chem..

[25] van Eldik R., Dasgupta T.P., Harris G.M. (1975). Kinetics and mechanism of aquation and formation reactions of carbonato complexes. IX. Aquation of α- and β-cis carbonato(ethylenediaminediacetato)cobaltate(III) ions in strongly acidic solution. Inorg. Chem..

[26] Jacewicz D., Banecki B., Dąbrowska A., Woźniak M., Chmurzyński L. (2004). Kinetics and mechanisms of the CO_2_ and SO_2_ uptake by coordinate ion, *cis*-[Cr(C_2_O_4_)(L-L)(OH_2_)_2_]^+^ {(L-L) = methyl 3-amino-2,3-dideoxy-α-D-*arabino*-hexopyranoside} studied by *stopped-flow* spectrophotometry. Inorg. Chim. Acta.

[27] Palmer D.A., Dasgupta T.P., Kelm P. (1978). Kinetics and mechanism of aquation and formation reactions of *cis*-carbonatobis(oxalato)chromate(III) ion in aqueous solution. Inorg. Chem..

[28] Buckingham D.A., Clark C.R. (1993). Acid-catalyzed hydrolysis of the carbonatobis(ethylenediamine)cobalt(1^+^) ion revisited. Inorg. Chem..

[29] Palmer D.A., Dasgupta T.P., Kelm H. (1978). Kinetics and mechanism of aquation and formation reactions of *cis*-carbonatobis(oxalato)chromate(III) ion in aqueous solution. Inorg. Chem..

[30] Jacewicz D., Łapińska A., Dąbrowska A., Chmurzyński L. (2006). A *Stopped-flow* study on the kinetics and mechanizm of CO_2_ uptake by the *cis*-[Cr(1,10-phenantroline)_2_(OH_2_)_2_]^3+^ complex ion. Trans. Met. Chem..

[31] Johanson M.L., Correira J.J., Yphantis D.A., Halvorson H.R. (1981). Analysis of data from the analytical ultracentrifuge by nonlinear least-squares techniques. Biophys. J..

[32] Nagle J.F., Parodi L.A., Lozier R.H. (1982). Procedure for testing kinetic models of the photocycle of bacteriorhodopsin. Biophys. J..

